# Improving Free Radical Scavenging Activity of Soy Isoflavone Glycosides Daidzin and Genistin by 3′-Hydroxylation Using Recombinant *Escherichia coli*

**DOI:** 10.3390/molecules21121723

**Published:** 2016-12-15

**Authors:** Chien-Min Chiang, Dong-Sheng Wang, Te-Sheng Chang

**Affiliations:** 1 Department of Biotechnology, Chia Nan University of Pharmacy and Science, No. 60, Sec. 1, Erh-Jen Rd., Jen-Te District, Tainan 71710, Taiwan; 2 Department of Biological Sciences and Technology, National University of Tainan, Tainan 70005, Taiwan; afg459326@yahoo.com.tw (D.-S.W.); mozyme2001@gmail.com (T.-S.C.)

**Keywords:** antioxidant, biotransformation, daidzin, genistin, hydroxylation

## Abstract

The present study describes the biotransformation of a commercially available crude extract of soy isoflavones, which contained significant amounts of the soy isoflavone glycosides daidzin and genistin, by recombinant *Escherichia coli* expressing tyrosinase from *Bacillus megaterium*. Two major products were isolated from the biotransformation and identified as 3′-hydroxydaidzin and 3′-hydroxygenistin, respectively, based on their mass and nuclear magnetic resonance spectral data. The two 3′-hydroxyisoflavone glycosides showed potent 2,2-diphenyl-1-picrylhydrazyl free radical scavenging activity with IC_50_ values of 7.4 and 9.8 μM for 3′-hydroxydaidzin and 3′-hydroxygenistin, respectively. The free radical scavenging activities of the two 3′-hydroxyisoflavone glycosides were, respectively, 120 and 72 times higher than the activity of their precursors, daidzin and genistin, and were also stronger than the activity of ascorbic acid, which showed an IC_50_ value of 15.1 μM. This is the first report of the bio-production and potential antioxidant applications of both 3′-hydroxydaidzin and 3′-hydroxygenistin.

## 1. Introduction

Isoflavones are dietary phytoestrogens occurring naturally in some plants, specifically in legumes, such as soybeans [1]. Two major isoflavones found in soybean are daidzin and genistin, which are the glycoside conjugates of daidzein and genistein, respectively. They account for more than 0.1% (*w/w*) of the dry weight of soybeans. In the past few decades, these isoflavones have been under intense investigation due to their possible role in preventing certain hormone-dependent and other diseases, including breast and prostate cancers, osteoporosis, and cardiovascular diseases [2]. In recent years, biotransformation of isoflavones using either wild-type or genetically engineered microorganisms has also been of interest because the bioactivity of isoflavones dramatically alters as the structure is altered.

*ortho*-Hydroxylation of the soy isoflavone aglycones daidzein and genistein is one of the major biotransformation reactions during the preparation of some fermented soybean foods using wild microorganisms such as *Bacillus*, *Aspergillus*, and *Rhizopus*. Many *ortho*-hydroxylated soy isoflavones, including 6-hydroxydaidzein, 6-hydroxygenistein, 8-hydroxydaidzein, 8-hydroxygenistein, 3′-hydroxydaidzein, and 3′-hydoxygenistein, have been isolated from fermented soybean foods and identified with multiple bioactivities [3]. Recently, some genetically engineered microorganisms were developed to catalyze *ortho*-hydroxylation of daidzein and/or genistein [3,4,5,6,7]. Most researchers used the cytochrome P450 oxidation system to develop the genetically engineered microorganisms and perform the biotransformation. However, Lee et al. used recombinant *Escherichia coli* expressing tyrosinase from *Bacillus megaterium* to catalyze 3′-hydroxylation of daidzein and genistein in the presence of borate and ascorbic acid [7]. They demonstrated that the biotransformation system using the recombinant *E. coli* showed both high productivity and high conversion yield in the biotransformation of daidzein and genistein into their 3′-hydroxylated derivatives. Therefore, application of the system to other molecules is worth exploring. In the present study, a crude extract of soy isoflavones, which contained primarily the soy isoflavone glycosides daidzin and genistin, was biotransformed using recombinant *E. coli* and the biotransformation products were isolated and their free radical scavenging activity was assayed using the 2,2-diphenyl-1-picrylhydrazyl (DPPH) method.

## 2. Results

### 2.1. Construction of Recombinant E. coli Expressing Tyrosinase from B. megaterium

**Figure 1:**
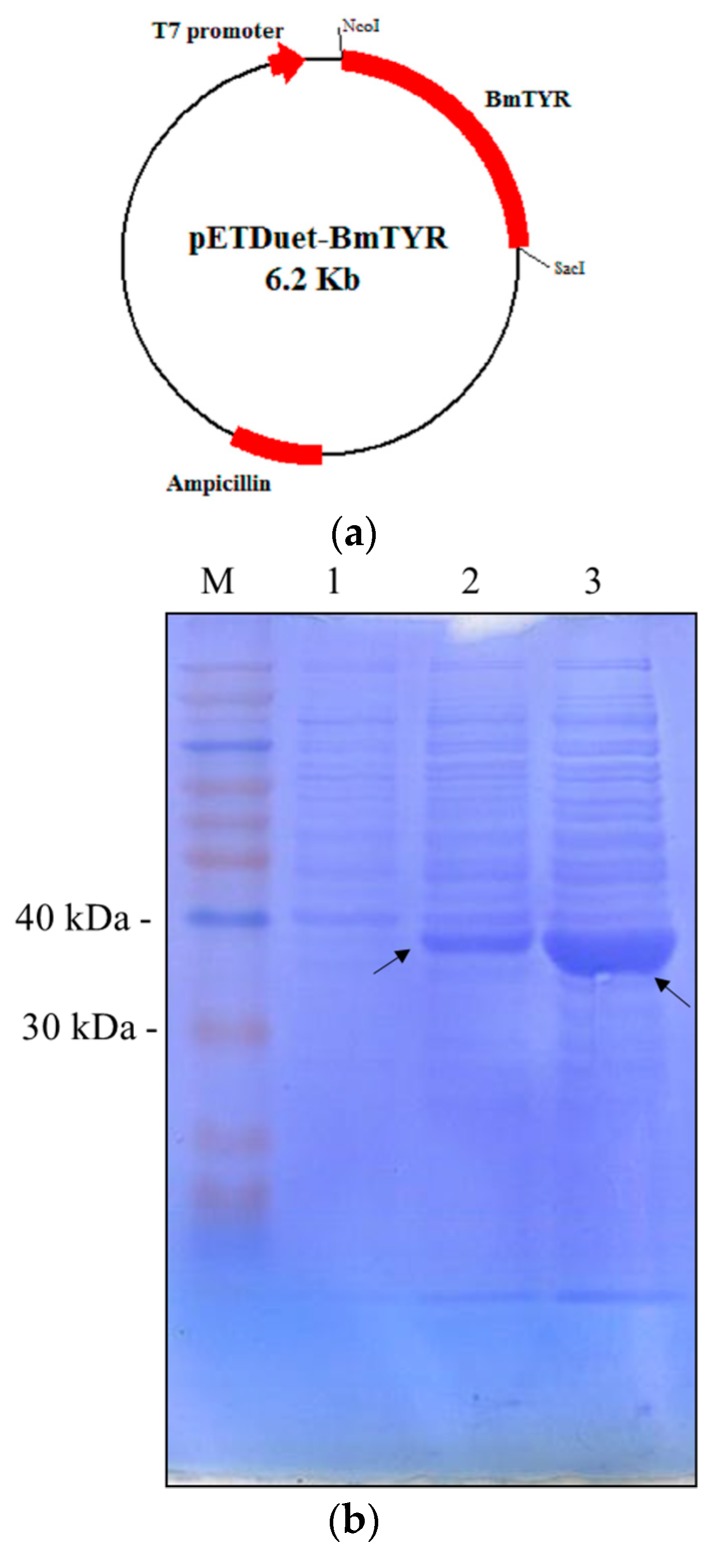

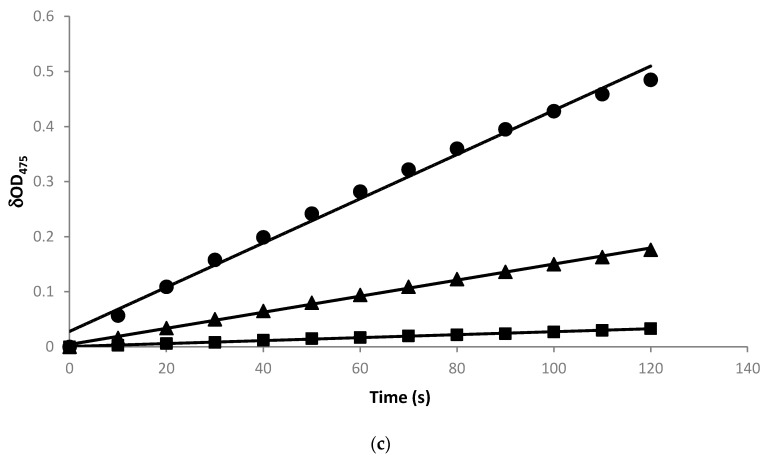
Construction and expression of *B. megaterium* tyrosinase in *E. coli*. (**a**) Map of the expression vector pETDuet-BmTYR. (**b**) Sodium dodecyl sulfate polyacrylamide gel electrophoresis (SDS-PAGE) of recombinant *E. coli* harboring pETDuet-BmTYR. Crude proteins from whole cell lysis with 0 h induction (lane 1), 4 h induction (lane 2), and 24 h induction (lane 3) were separated using SDS-PAGE. Arrows indicate the expressed tyrosinase. M represents the molecular weight markers. (**c**) Tyrosinase activity assay using l-DOPA as substrate. Either 20 μg (─￭─), 100 μg (─▲─), or 400 μg (─●─) of the lyophilized recombinant cells was mixed with 2.5 mM of l-DOPA in 1 mL phosphate-buffered saline (PBS, pH 6.8) and the resulting dopachrome was monitored at 475 nm with a spectrophotometer. One unit of tyrosinase activity is the amount of cells that produces one μmole of dopachrome (absorption efficiency ε = 3600 cm^−1^M^−1^) per minute from the reaction.

The tyrosinase gene from *B. megaterium* was amplified by polymerase chain reaction (PCR) and subcloned into pETDuet-1^TM^ (Novagen, Madison, WI, USA) to form the expression vector pETDuet-BmTYR (Figure 1a). The recombinant *E. coli* harboring the expression vector was confirmed to produce considerable tyrosinase protein (36 kDa) after isopropyl-β-d-thiogalactopyranoside (IPTG) induction (Figure 1b). In addition, the tyrosinase activity of the recombinant cells was determined using l-3,4-dihydroxyphenylalanine (l-DOPA) as a substrate. The results showed that 1 mg of the lyophilized cell mass of the recombinant *E. coli* contained 0.25 units of tyrosinase activity (Figure 1c). Therefore, the recombinant cells were used for the biotransformation study.

### 2.2. Biotransformation of a Soy Isoflavone Crude Extract by the Recombinant E. coli and Purification and Identification of the Biotransformation Products

**Figure 2:**
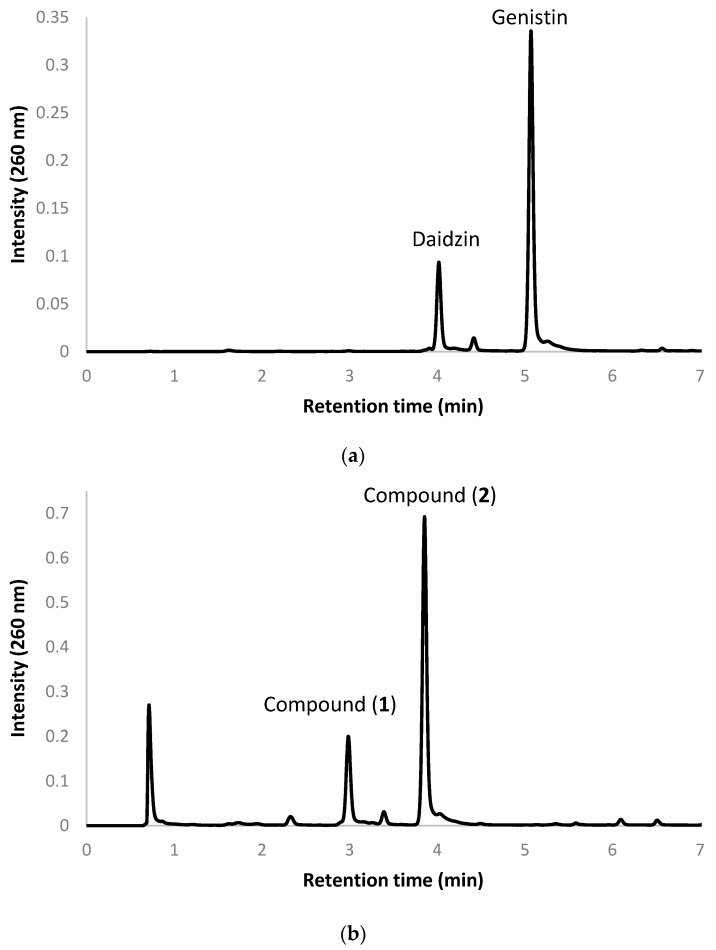
(**a**) UPLC profiles of the commercial soy isoflavone crude extract, and (**b**) the biotransformation after reaction with the recombinant *E. coli* for 1.5 h. The detailed protocols for biotransformation and UPLC are described in the Materials and Methods section.

The present study used a commercial soy isoflavone crude extract. The crude extract contained 40% (*w/w*) soy isoflavones, of which daidzin and genistin were the major components. Figure 2a shows the ultra-performance liquid chromatography (UPLC) profile of the commercial soy isoflavone extract. In the figure, the two major components daidzin and genistin appear at retention times of 4.02 and 5.11, respectively. For studying biotransformation, 20 U of the lyophilized recombinant *E. coli* was mixed with 0.2 g of the soy isoflavone crude extract in a 100 mL reaction mixture containing 500 mM of borate (pH 9.0) and 10 mM of ascorbic acid. After a reaction time of 1.5 h, the products’ profiles were determined with UPLC to check whether the substrate could be converted by the recombinant cells. As shown in Figure 2b, nearly all of the precursor daidzin and genistin disappeared, and two major metabolites, compounds (**1**) and (**2**), appeared as new peaks at retention times of 3.00 and 3.88 min, respectively, in the profile of the biotransformed extract at 1.5 h of incubation. The metabolites were further isolated by using a preparative high-performance liquid chromatography (HPLC) method and were identified by using spectrophotometric methods. Compound (1) showed an [M + H]^+^ ion peak at *m*/*z* 433 in the electrospray ionization mass (ESI-MS) spectrum, corresponding to the molecular formula C_21_H_20_O_10_. Then, ^1^H and ^13^C nuclear magnetic resonance (NMR) were performed to elucidate the structure. The assignment of the ^1^H- and ^13^C-NMR signals was conducted according to heteronuclear single quantum coherence (HSQC), heteronuclear multiple bond connectivity (HMBC), distortionless enhancement by polarization transfer (DEPT), nuclear overhauser effect spectroscopy (NOESY), and correlation spectroscopy (COSY). 

The following data were collected for compound (**1**): ^1^H-NMR (DMSO-*d*_6_, 500 MHz) δ: 8.35 (1H, s, H-2), 8.04 (1H, d, *J* = 8.9 Hz, H-5), 7.22 (1H, d, *J* = 2.4 Hz, H-8), 7.18 (1H, dd, *J* = 8.9, 2.4 Hz, H-6), 7.03 (1H, d, *J* = 2.1 Hz, H-2′), 6.83 (1H, dd, *J* = 8.1, 2.1 Hz, H-6′), 6.76 (1H, d, *J* = 8.1 Hz, H-5′), 5.10 (1H, d, *J* = 6.8 Hz, Glc H-1), 3.72–3.48 (2H, m, Glc H-6), 3.45 (1H, m, Glc H-5), 3.36 (1H, m, Glc H-3), 3.30 (1H, m, Glc H-2), 3.18 (1H, m, Glc H-4); ^13^C-NMR (DMSO-*d*_6_, 125 MHz) δ: 174.7 (C-4, C=O), 161.3 (C-7), 156.9 (C-9), 153.2 (C-2), 145.5 (C-4′), 144.9 (C-3′), 126.9 (C-5), 123.8 (C-3), 122.7 (C-1′), 119.8 (C-6′), 118.5 (C-10), 116.6 (C-2′), 115.5 (C-5′), 115.4 (C-6), 103.4 (C-8), 100.0 (Glc C-1), 77.2 (Glc C-5), 76.5 (Glc C-3), 73.1 (Glc C-2), 69.6 (Glc C-4), and 60.6 (Glc C-6). Based on these spectral data and with the comparison of ^1^H-NMR and ^13^C-NMR data in the literature [8], compound (**1**) was characterized as 3′-hydroxydaidzin. 

Compound (**2**) was obtained as a pale yellow powder and showed an [M + H]^+^ ion peak at *m*/*z* 449 and the ^1^H-NMR (DMSO-*d*_6_, 700 MHz) δ: 8.38 (1H, s, H-2), 7.02 (1H, d, *J* = 2.2 Hz, H-2′), 6.84 (1H, dd, *J* = 8.1, 2.2 Hz, H-6′), 6.80 (1H, d, *J* = 8.1 Hz, H-5′), 6.71 (1H, d, *J* = 2.2 Hz, H-8), 6.47 (1H, d, *J* = 2.2 Hz, H-6), 5.06 (1H, d, *J* = 7.4 Hz, Glc H-1), 3.70–3.45 (2H, m, Glc H-6), 3.44 (1H, m, Glc H-5), 3.33 (1H, m, Glc H-3), 3.28 (1H, m, Glc H-2), 3.18 (1H, m, Glc H-4). ^13^C-NMR (DMSO-*d*_6_, 176 MHz) δ: 180.6 (C-4), 163.1 (C-7), 161.8 (C-5), 157.3 (C-9), 154.7 (C-2), 145.7 (C-4′), 145.0 (C-3′), 122.8 (C-1′), 121.5 (C-3), 120.1 (C-6′), 116.6 (C-2′), 115.5 (C-5′), 106.2 (C-10), 99.9 (C-1”), 99.7 (C-6), 94.6 (C-8), 77.3 (Glc C-3), 76.4 (Glc C-5), 73.2 (Glc C-2), 69.7 (Glc C-4), and 60.7 (Glc C-6). By comparing these data with the values in the literature [9], compound (**2**) was identified as 3′-hydroxygenistin.

**Figure 3:**
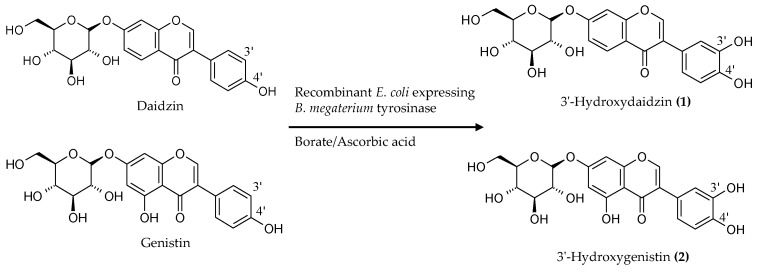
Diagram of the biotransformation of the soy isoflavone glycosides daidzin and genistin by the recombinant *E. coli* expressing *B. megaterium* tyrosinase.

The results revealed that the recombinant *E. coli* expressing *B. megaterium* tyrosinase could catalyze 3′-hydroxylation of the soy isoflavone glycosides daidzin and genistin. Figure 3 shows the diagram of the biotransformation of the soy isoflavone glycosides daidzin and genistin by the recombinant *E. coli* expressing *B. megaterium* tyrosinase.

### 2.3. Radical Scavenging Activity of the Biotransformation Products

**Figure 4:**
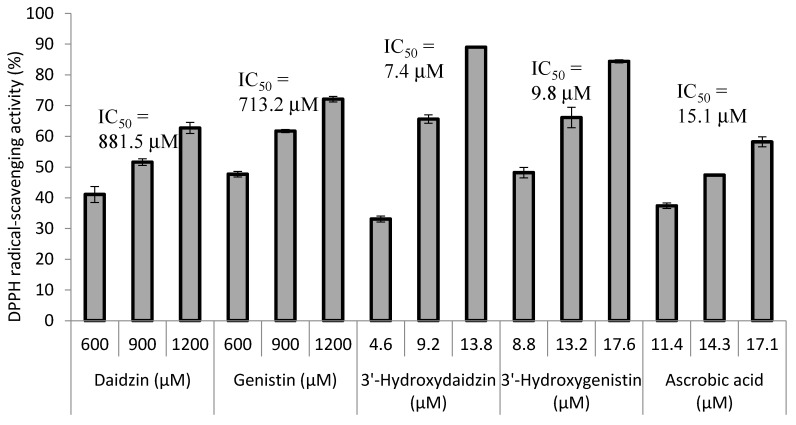
DPPH radical-scavenging activity of soy isoflavones and ascorbic acid. The DPPH scavenging activity was determined as previously described [10]. The IC_50_ values represent the concentrations required for 50% DPPH radical-scavenging activity. The mean (*n* = 3) is shown, and the standard deviations are represented by error bars.

The antioxidant activity of the soy isoflavones was determined using the DPPH free radical scavenging assay system. The results are shown in Figure 4. The two precursors, daidzin and genistin, did not show potent free radical scavenging activity (IC_50_ values of 881.5 and 713.2 μM, respectively). However, after the 3′-hydroxylation modification, the radical scavenging activity of the 3′-hydroxylated derivatives was 120 and 72 times more potent than the activity of the precursors, with IC_50_ values of 7.4 and 9.8 μM for 3′-hydroxydaidzin and 3′-hydroxygenistin, respectively. The IC_50_ values of the 3′-hydroxylated derivatives were also lower than the values of ascorbic acid (IC_50_ = 15.1 μM), which is the gold standard antioxidant.

## 3. Discussion

Tyrosinases (EC 1.14.18.1) catalyze the oxidation of both monophenols (cresolase or monophenolase activity) and *o*-diphenols (catecholase or diphenolase activity) into reactive *o*-quinones; the enzyme is widely distributed in nature [11]. In our previous searches for tyrosinase inhibitors, we used tyrosinase from mushroom (*Agaricus bisporus*) as an enzyme source and discovered that both the soy isoflavone aglycone daidzein and the glycoside daidzin were mushroom tyrosinase inhibitors [12]. Moreover, based on the kinetic data, the two inhibitors were proven to be competitive. The results implied that both daidzein and daidzin could bind to the active site of mushroom tyrosinase. Recently, Lee et al. used tyrosinase from *B. megaterium* to catalyze 3′-hydroxylation of the soy isoflavone aglycones daidzein and genistein [7]. Based on our previous finding on mushroom tyrosinase, we suggested that the soy isoflavone glycosides daidzin and genistin might also bind to the active site of the tyrosinase from *B. megaterium* and might even be catalyzed by the enzyme. Our present results confirmed the suggestion and demonstrated that recombinant *E. coli* expressing *B. megaterium* tyrosinase could catalyze 3′-hydroxylation of soy isoflavone glycosides daidzin and genistin. 

Moreover, the present study is also the first report of the bio-productions of 3′-hydroxydaidzin and 3′-hydroxygenistin. Hydroxylation of aromatics by chemical synthesis is difficult and involves several reaction steps. Biotransformation by resting cells of microorganism is simpler, less expensive, and more convenient. Because the two soy isoflavone glycosides daidzin and genistin are the major components of soy isoflavones, a commercial soy isoflavone crude extract could be directly applied to the bio-production process in the present study without any pretreatments such as deglycosylation, and the biotransformation was done in one step. The soy isoflavone crude extract was commercially available by the kilogram, so the bio-production process in the present study could be applied on an industrial scale. We thus believe that the reported bio-production process for the two 3′-hydroxylated soy isoflavone glycosides has high potential for industrial applications.

In contrast to soy isoflavones, which exist abundantly in soybeans, *ortho*-hydroxyisoflavones exist rarely in nature. Most *ortho*-hydroxyisoflavones are biotransformed products from isoflavones by microorganisms. The microbial enzymes, which could catalyze the biotransformation, include heme-containing monooxygenase (cytochrome P450 protein; CYP) [3], non-heme, flavin-dependent monooxygenase [6], and the tyrosinase used in the present study [7]. The *ortho*-hydroxylation of the precursors might occur at the C-6, C-8, or C-3′ position based on the regioselectivity of the enzyme. Among the discovered microbial enzymes with *ortho*-hydroxylation activity, enzymes with 3′-hydroxylation activity are most abundant, including most bacterial CYPs, the non-heme, flavin-dependent monooxygenase and the tyrosinase. Only fungal CYP57B3 from *A. oryzae* and bacterial CYP from *Nocardia farcinica* catalyzed C-6 and/or C-8 *ortho*-hydroxylation of daidzein [4,13]. In the view of productivity, the tyrosinase produced over 100-fold higher productivity of 3′-hydroxyisoflavones than other enzymes [7]. However, the tyrosinase did not catalyze C-6 nor C-8 *ortho*-hydroxylation. Therefore, improving productivity of C-6 or C-8 *ortho*-hydroxylation needs further study in the future. 

Free radicals have been suggested as potentially important causative agents of several human diseases. Soy isoflavones can remove free radicals from the human body, thereby potentially preventing aging, cardiovascular diseases, and cancer [2]. The free radical scavenging activity of isoflavones is closely associated with the structures of the isoflavones [14]. Both number and position of the hydroxyl groups on the skeleton of the isoflavones dramatically affect the free radical scavenging activity. The results of the present study showed that the produced 3′-hydroxylated soy isoflavone derivatives 3′-hydroxydaidzin and 3′-hydroxygenistin possessed more potent DPPH free radical scavenging activity than the precursors daidzin and genistin. Kowalska et al. have reported the potent antioxidant activity of 3′-hydroxydaidzin before [8]. However, the present study was the first report about the potent antioxidant activity of 3′-hydroxygenistin. The results are consistent with previous findings that flavonoids with two hydroxyl groups at the 3′ and 4′ positions of the B-ring showed stronger antioxidant activity than flavonoids without or with single hydroxyl group [14]. In addition to our finding, Ye et al. isolated a *Trichoderma harzianum* and used the wild-type strain to catalyze the biotransformation of naringin (glycoside of flavanone naringein) into 3′-hydroxynaringin [15]. In addition, Liu et al. used the same strain to produce 3′-hydroxypuerarin from puerarin (8-C glycoside of isoflavone daidzein) [16]. In those two studies, the authors found that the produced 3′-hydroxylated derivatives possessed more potent free radical scavenging activity than their precursors [15,16]. Therefore, the results of the present study together with the results of the other described studies demonstrate that 3′-hydroxylation of flavonoid glycosides using either wild-type or genetically engineered microorganisms is a promising strategy for improving the free radical scavenging activity of flavonoid glycosides. On the other hand, Fussell et al. recently reported that catechol metabolites of estrogens induce redox cycling and generate reactive oxygen species (ROS), including H_2_O_2_ and hydroxyl radicals in breast epithelial cells; redox cycling was not observed with the parent estrogens [17]. Although the produced 3′-hydroxyflavonoids in the present study possess higher free radical scavenging activity, if the resulting catechol moiety of the produced 3′-hydroxyflavonoids redox cycles and generates ROS in cells needs further study.

## 4. Materials and Methods

### 4.1. Microorganisms and Chemicals

We purchased *B. megaterium* BCRC 10608 from the Bioresources Collection and Research Center (BCRC, Food Industry Research and Development Institute, Hsinchu, Taiwan). The expression system containing both *E. coli* BL21 (DE3) and vector pETDuet-1^TM^ was obtained from Novagen. IPTG, l-DOPA, dimethyl sulfoxide (DMSO), daidzin, and genistin were purchased from Sigma (St. Louis, MO, USA). The soy isoflavone crude extract containing 40% (*w/w*) soy isoflavones and 60% (*w/w*) of mixture of soy protein, carbohydrate, and lipid was bought from Glory Biotechnology Company (Chiayi, Taiwan). The other reagents and solvents used were of high quality and were commercially available.

### 4.2. Expression of B. megaterium Tyrosinase in E. coli

The genomic DNA of *B. megaterium* was isolated using the commercial kit Geno *Plus*^TM^ (Viogene, Taipei, Taiwan). The *B. megaterium* tyrosinase gene (GenBank protein database accession number KGJ77254) was amplified from the genomic DNA by PCR with primer set of forward: 5′-gggCCATGGgtaacaagtacagagttagaa-3′; reverse: 5′-gggGAGCTCttatgatgaacgttttgattttc-3′. Restriction enzyme recognizing sites were designed at the forward primer (NcoI, CCATGG) and the reverse primer (SacI, GAGCTC). The amplified tyrosinase gene was subcloned into the pETDuet-1™ vector through the NcoI and SacI sites to obtain the expression vector pETDuet-BmTYR (Figure 1a). The expression vector was transformed into *E. coli* BL21 (DE3) via electroporation to obtain the recombinant *E. coli*. The recombinant *E. coli* was cultivated in 100 mL of Luria-Bertani (LB) medium containing 50 μg/mL of ampicillin with 200 rpm shaking at 37 °C [7]. When the optical density at 600 nm reached 0.6, 0.1 mM IPTG and 1 mM CuSO_4_ were added to induce expression of tyrosinase. The cells were continuously cultured in an incubator at 18 °C for another 24 h. At the end of the cultivation, cells were harvested by centrifugation at 5000 rpm and 4 °C and washed once with 100 mL of PBS. Cell pellets from the washed cells were lyophilized by a freeze dryer. Finally, 160 mg of lyophilized recombinant cell mass was obtained and used for the biotransformation. 

### 4.3. Tyrosinase Activity Assay

Tyrosinase activity was measured as previously reported [12]. First, 20 mg of lyophilized recombinant cell mass was dissolved in 1 mL of PBS and equivalent amount of 20, 100, or 400 μg of the lyophilized recombinant cell mass was mixed with 1 mL of 2.5 mM l-DOPA (dissolved in PBS pH 6.8) and then incubated at 25 °C for 2 min. Dopachrome formation in each reaction was monitored with a UV-VIS spectrophotometer (U-5100, Hitachi, Japan) at 475 nm.

### 4.4. Biotransformation and UPLC Analysis

The biotransformation system was operated according to the report by Lee et al. [7] with minor modifications. First, 20 mg of the lyophilized recombinant cells was added into 100 mL of reaction mixture containing 500 mM borate (pH 9.0), 10 mM ascorbic acid, and 0.2 g of the soy isoflavone crude extract (100 mg/mL in DMSO). The reaction mixture was then set in an incubator at 50 °C and shaken at 200 rpm for 1.5 h. At the end of the reaction, 20 mL of 1 M HCl and 120 mL of MeOH were added to stop the reaction. The mixture was analyzed with a UPLC system (ACQUITY UPLC H-Class, Waters, Milford, MA, USA) equipped with an analytic C18 reversed-phase column (Acquity UPLC BEH C18, 1.7 μm, 2.1 i.d. × 100 mm, Waters, Milford, MA, USA). The operation conditions included a gradient elution using water (A) containing 1% (*v/v*) acetic acid and methanol (B) with a linear gradient for 6 min with 20% to 50% B and for another 1 min with 50% to 60% B at a flow rate of 0.3 mL/min, an injection volume of 0.2 μL, and detection of the absorbance at 260 nm.

### 4.5. Purification and Identification of Biotransformation Products

The 200 mL of the reaction mixture obtained from the above biotransformation was filtered with a 0.22 μm nylon membrane under vacuum. Then, the filtrate was injected into a preparative YoungLin HPLC system (YL9100, YL Instrument, Gyeonggi-do, Korea) equipped with a quaternary pump (YL9110, YL Instrument, Gyeonggi-do, Korea), a UV/Vis detector (YL9120, YL Instrument), and a preparative C18 reversed-phase column (Inertsil, 10 μm, 20.0 i.d. × 250 mm, ODS 3, GL Sciences, Eindhoven, The Netherlands) for purification of the biotransformation products. The operational conditions for the preparative HPLC analysis included a gradient elution using water (A) containing 1% (*v/v*) acetic acid and methanol (B) with a linear gradient for 25 min with 25% to 50% B at a flow rate of 15 mL/min, and detection of the absorbance at 260 nm. The injection volume was 10 mL. The elution corresponding to the peak of the metabolite in the UPLC analysis was collected, condensed under a vacuum, and then lyophilized by freeze-drying. Finally, 17.5 mg of compound (**1**) and 16.0 mg of compound (**2**) were obtained, and the structures of the compounds were confirmed with NMR and mass spectral analysis. The mass analysis was performed using a Finnigan model LCQ Duo mass spectrometer (ThermoQuest Corp., San Jose, CA, USA) with electrospray ionization (ESI). ^1^H- and ^13^C-NMR, DEPT, COSY, NOESY, HSQC, and HMBC spectra were recorded on Bruker AV-500 and AV-700 NMR spectrometers (Bruker Corp., Billerica, MA, USA). Standard pulse sequences and parameters were used for the NMR experiments and all chemical shifts were reported in parts per million (ppm, δ). According to the mass and NMR spectral, the purity of the two purified compounds were estimated to be 95%.

### 4.6. DPPH Free Radical Scavenging Activity Assay

The assay was carried out as previously described [10] with minor modifications. The isoflavones (dissolved in DMSO) were added to the DPPH (1 mM) solution to a final volume of 1 mL. After 30 min of reaction, the absorbance of the reaction mixture was read at 517 nm with a UV-Vis spectrophotometer (U-5100, Hitachi, Japan). Ascorbic acid (dissolved in DMSO) was used as a positive antioxidant standard. The DPPH radical scavenging activity was calculated as DPPH radical scavenging activity = (OD_517_ of control reaction − OD_517_ of reaction)/(OD_517_ of control reaction). 

## 5. Conclusions

The present study is the first to prove that tyrosinase from *B. megaterium* could catalyze 3′-hydroxylation of the soy isoflavone glycosides daidzin and genistin. Moreover, this is also the first report of the bio-production of 3′-hydroxydaidzin and 3′-hydroxygenistin. The produced 3′-hydroxylated soy isoflavone derivatives possessed more potent DPPH free radical scavenging activity than their precursors, daidzin and genistin. The results of the present study provide insight into the production of both 3′-hydroxydaidzin and 3′-hydroxygenistin and their potential antioxidant applications.
